# Unveiling the Metaverse: A survey of user perceptions and the impact of usability, social influence and interoperability

**DOI:** 10.1016/j.heliyon.2024.e31413

**Published:** 2024-05-16

**Authors:** Mousa Al-kfairy, Ayham Alomari, Mahmood Al-Bashayreh, Omar Alfandi, Mohammad Tubishat

**Affiliations:** a Zayed University, College of Technological Innovation, United Arab Emirates; b Faculty of Information Technology, Applied Science Private University, Amman, Jordan

**Keywords:** Metaverse user perception, Social influence, Usability, Interoperability, Technology acceptance, Review paper

## Abstract

This review explores the Metaverse, focusing on user perceptions and emphasizing the critical aspects of usability, social influence, and interoperability within this emerging digital ecosystem. By integrating various academic perspectives, this analysis highlights the Metaverse's significant impact across various sectors, emphasizing its potential to reshape digital interaction paradigms. The investigation reveals usability as a cornerstone for user engagement, demonstrating how social dynamics profoundly influence user behaviors and choices within virtual environments. Furthermore, the study outlines interoperability as a paramount challenge, advocating for establishing unified protocols and technologies to facilitate seamless experiences across disparate Metaverse platforms. It advocates for the adoption of inclusive, ergonomically oriented designs aimed at enhancing user participation. It addresses the ethical and societal challenges posed by the Metaverse, including concerns related to digital harassment, invasive marketing practices, and breaches of privacy. Additionally, the review identifies existing gaps in the literature, particularly regarding the Metaverse's implications for healthcare, its impact on educational outcomes, and the urgent need for empirical data concerning its long-term effects on user psychology and behavior.

By providing a comprehensive synthesis of the current understanding of user experiences and challenges within the Metaverse, this paper contributes to the academic dialogue, laying the groundwork for future research initiatives. It aims to steer the development of the Metaverse towards a trajectory that is ethically sound, socially responsible, inclusive, and aligned with societal expectations, thereby fostering a digital realm that upholds the highest standards of integrity and inclusivity.

## 1 Introduction

In the dynamic and ever-expanding field of computer science and information systems, the emergence of the Metaverse has marked a paradigm shift, capturing the imagination and scholarly attention of experts worldwide. This paper presents a comprehensive survey of existing literature, exploring the complex landscape of the Metaverse. Unlike a systematic review or meta-analysis, this survey aims to compile and combine various academic perspectives, offering a panoramic view of the Metaverse's evolving role and significance in the digital domain. Our approach is rooted in the disciplines of computer science and information systems, reflecting the Metaverse's profound implications in these areas [1,2].

The concept of the Metaverse, which originated in the realm of science fiction, has now grown into a rapidly expanding area of research and development, transcending its fictional roots to establish itself as a complex, multi-faceted digital ecosystem. Its applications and implications stretch across a spectrum of areas, from gaming and entertainment to critical sectors like education, healthcare, and business [[3], [4], [5]]. In the sphere of computer science, the Metaverse is not just a technological novelty but a frontier of innovation, integrating advanced technologies such as virtual and augmented reality, artificial intelligence, and blockchain. These

technologies are not standalone phenomena but are interwoven within the fabric of the Metaverse, creating a unique digital experience [6].

The significance of the Metaverse in the context of information systems is equally profound. It represents a new model of user interaction, data exchange, and digital ecosystems, challenging traditional paradigms and offering novel avenues for exploration and application. The Metaverse's development and expansion raise critical questions about user engagement, system interoperability, and the social dynamics within virtual environments. These aspects are pivotal in shaping the future trajectory of the Metaverse and are of paramount interest to researchers, developers, and practitioners in the field.

Guided by this context, our survey paper is structured to address three fundamental research questions that capture the essence of user interaction and system functionality within the Metaverse.

1. What are users' perceptions regarding the usability of the Metaverse? How does it influence their engagement and satisfaction?
2. How do users perceive the interoperability of different Metaverse platforms? Are there preferences or significant challenges identified?
3. In what ways does the social influence within the Metaverse impact user behavior and perceptions?

These questions are specifically designed to analyze the Metaverse's user-centric features, examining how usability, interoperability, and social influence converge to define user experiences. The answers to these questions are crucial for advancing our understanding of the Metaverse, particularly in the realms of computer science and information systems. They provide insights into the design, development, and deployment of future technologies within the Metaverse, ensuring that these innovations are aligned with user needs and preferences. This survey, therefore, serves a dual purpose. Firstly, it aims to consolidate the current state of knowledge about the Metaverse, providing a comprehensive overview for academics and practitioners alike. Secondly, it seeks to identify gaps in the existing literature, setting the stage for future research directions that can propel the field forward. By exploring the intersection of technology, user experience, and social dynamics, this paper contributes to the ongoing discourse in the field, offering a foundation for the responsible and user-centric

Development of the Metaverse.

In conclusion, this survey article provides a comprehensive examination of the Metaverse while situating itself at the intersection of computer science and information systems. It seeks to promote a better comprehension of this digital phenomenon by highlighting its opportunities, difficulties, and future directions for study and advancement.

In order to achieve the study aims, the next section discusses the related work, and then we introduce the Methodology used for conducting this review. After that, we discuss the study themes, Usability, Interoperability, and Social Influence. Then, the findings and implications are discussed. The final section concludes.

## 2 Related work

Several studies have investigated users' perceptions of the Metaverse and its impact on various domains (see Table 1 that summarises Metaverse study aspects). The Metaverse, a collective digital space, merges augmented reality (AR), virtual reality (VR), and the internet, providing an immersive experience for its users [7,8]. As the next step in the internet's evolution, the Metaverse is rapidly becoming influential across several industries, with education and healthcare being particularly notable.

**Table 1:** Summary of Metaverse studies.

Study Reference	Topics Discussed	Outcome/Conclusion
[9,10]	Technology adoption	Factors like performance expectations, ease of use, and trust are crucial for technology adoption. Younger participants are more inclined towards Metaverse technology.
[11,12]	Integration of advanced tech in education	Technologies can enhance learning experiences. VR provides an immersive experience especially beneficial in healthcare education.
[13,14]	User perceptions	Importance of UX design, visualization, and involving users in decision-making.
[15]	Academic research potentials in the Metaverse	AI, blockchain, and virtual reality have significant implications for marketing in the metaverse.
[[16], [17], [18]]	Security	Emphasize on privacy regulations, stringent security measures, and the need for effective public policy to regulate the Metaverse.
[19]	Adoption	Importance of understanding user perception over just technical features.
[[20], [21], [22]]	User experience, perception	Highlights potential of Metaverse in online education and remote training.
[20,21,[23], [24], [25], [26]]	Metaverse's potential across various industries	Emphasizes the need for more research to understand the transformative impact of the Metaverse.
[[27], [28], [29]]	Consumer experience, social presence, and immersive learning in Metaverse	Importance of service quality, immersive experiences, and the influence of devices on social presence.
[24,[30], [31], [32], [33]]	User perceptions in educational settings	Highlights the effectiveness and potential of Metaverse and AR technologies in improving learning outcomes.

Regarding users' perceptions of the Metaverse's usability, studies have shown that several factors influence user engagement and satisfaction.

Studies like [7], found that perceived usefulness, ease of use, and social norms shape acceptance and satisfaction. Additionally, demographic variables, such as age, play a role in this acceptance. Younger users, for instance, seem more receptive to adopting Metaverse technologies [34]. Furthermore, the user experience (UX) is highlighted as pivotal for the Metaverse's success, with research emphasizing the importance of good UX design and suitable visualization techniques [13,14].

In terms of interoperability, user perceptions regarding different Metaverse platforms vary. While the literature doesn't provide a direct answer, it can be inferred that users are keen on platforms that allow customization, as demonstrated by Ref. [20]. Such preferences indicate a potential challenge for less flexible or user-centric platforms. Moreover, the dominance of specific platforms can influence user choices, pointing to the importance of seamless interactions across various Metaverse worlds, such as maze and escape room worlds [21].

Social influence within the Metaverse also significantly impacts user behavior and perceptions. The idea that social norms and factors influence the Metaverse's acceptance is evident in several studies [7,35]. Within these virtual spaces, user behaviors are likely affected by their peers and the broader community, suggesting that social elements are crucial drivers for engagement and active participation.

The social dynamics within the Metaverse and their impact on user behavior are acknowledged but not thoroughly dissected in current studies. While the influence of social norms and factors has been identified as.

Critical in Metaverse acceptance, there is limited knowledge of the intricate ways these social interactions and influences shape behaviour, decision-making, and overall user experiences. This presents a significant research gap that demands in-depth exploration to understand the complex interplay of social factors and individual perceptions in the virtual realm.

The existing research on user perception in the Metaverse has deepened our understanding of how users experience, view, and participate in virtual environments. However, there are still significant gaps in our knowledge, particularly regarding the impact of design elements and UX on overall satisfaction and sustained engagement. Additionally, while platform interoperability is recognized, there is a lack of research on user views of the challenges and preferences associated with different Metaverse platforms. Furthermore, elements such as usability, platform interoperability, and the inherent social dynamics inside the Metaverse are critical aspects that require further investigation and research [20,23,24].

This review paper identifies key areas that merit further investigation, including usability, platform compatibility, and the social impact of the metaverse. By addressing these gaps in our knowledge, we can develop a more comprehensive understanding of user perception and improve the overall metaverse experience. Our survey on the metaverse represents a substantial advancement in contemporary research. To the best of our knowledge, this investigation is deemed the inaugural endeavor addressing user perceptions of the metaverse concerning usability, platform interoperability, and social influence. Diverging from existing studies, our survey distinguishes itself by extending beyond a narrow technological focus. In contrast to predominantly technocentric approaches, we underscore the significance of comprehending individuals’ experiential dimensions within the metaverse.

## 3 Methodology

**Fig. 1:**
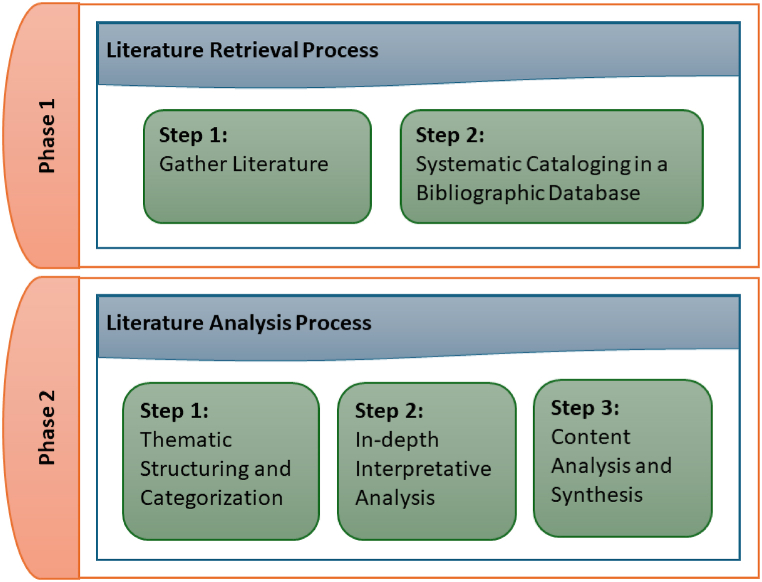
Research processes.

This study adopts a Narrative Literature Review (NLR) methodology meticulously designed to interrogate the multifaceted dimensions of user perceptions within the metaverse, focusing on usability, social influence, and interoperability. As depicted in Fig. 1, the methodology is bifurcated into two principal processes: the literature retrieval process and the literature analysis process. Each process is meticulously constructed to ensure a systematic exploration and synthesis of the extant literature, facilitating a nuanced understanding of the subject matter.

The first process (literature retrieval process) is constructed of two steps: the comprehensive literature collection and the systematic cataloging in a bibliographic database. The first step of the methodology is dedicated to the exhaustive collection of literature, spanning peer-reviewed articles, conference proceedings, and pertinent industry reports. This step ensures the acquisition of a diverse and comprehensive dataset, which is foundational for the ensuing analysis. The anticipated outcome is a rich repository of literature, providing a broad spectrum of insights into user perceptions, pivotal for underpinning subsequent analysis [36]. In this step, the research papers were obtained from several databases and publishers, including IEEE Xplore Digital Library, MDPI, Elsevier, IGI Global, AI Access Foundation, Taylor & Francis, and Wiley.

In the second step, the literature is systematically cataloged within a bibliographic database. This phase facilitates efficient management and retrieval of sources, ensuring a streamlined process for future reference and analysis. The resultant bibliographic repository significantly enhances the study's methodological rigor, laying the groundwork for a structured and accessible synthesis of literature [37].

The second process (literacy analysis process) is constructed of three steps: the thematic structuring and categorization, the in-depth interpretative analysis, and the content analysis and synthesis. The second process's first step is the thematic structuring and categorization of the literature under the direction of the predetermined units of analysis: user perceptions, metaverse, usability, social influence, and interoperability. The outcome of this phase is a coherent thematic framework, delineating the scope of the analysis and facilitating a focused narrative synthesis of the literature, thereby identifying prevailing themes, trends, and gaps within the field [38].

In the second step, an in-depth interpretative analysis of the literature follows, aiming to achieve a comprehensive understanding of the methodologies, findings, and theoretical contributions within the collected works. This meticulous engagement with the text is instrumental in identifying underlying patterns, theoretical constructs, and methodological nuances, enriching the review's narrative and contributing to a holistic synthesis of the literature [39].

**Fig. 2:**
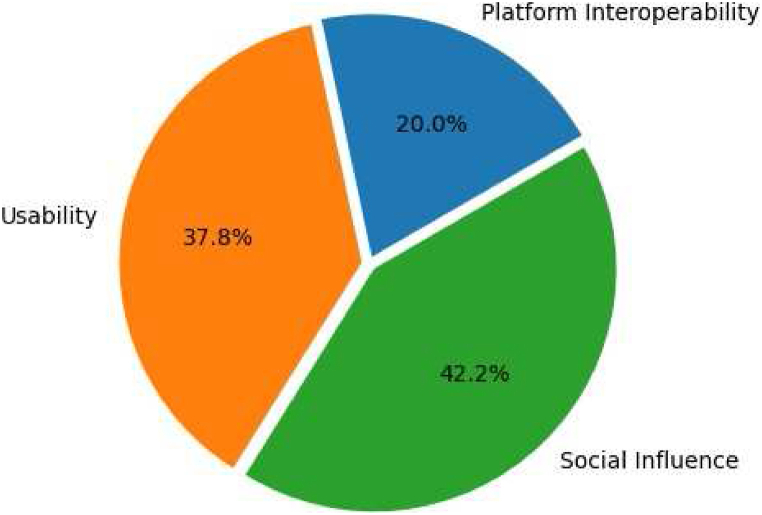
Publication per-area.

This is the third step, which uses a content analysis method to carefully look over the literature (Fig. 2) to find key insights, contradictions, and emerging patterns related to the metaverse's usability, social influence, and interoperability. This analytical rigor culminates in the synthesis of findings, highlighting significant gaps and delineating avenues for future research. This comprehensive synthesis not only contributes to the academic discourse but also proposes a scaffold for subsequent empirical investigations into the metaverse [40].

In summary, by adhering to a structured NLR methodology, this review endeavors to offer a comprehensive examination of user perceptions of the Metaverse, underpinned by a rigorous analytical framework. The systematic approach ensures the identification of significant themes, gaps, and theoretical constructs, advancing our understanding of the metaverse and paving the way for future research endeavors.

## 4 Users’ perception of the Metaverse

This section examines user perceptions of the Metaverse, analyzing various essential factors that influence users' experiences. It aims to investigate Platform Interoperability, Usability, and Social Influence to comprehensively understand their impact on users’ perceptions and engagement with the Metaverse. This study examines platform interoperability, which means that different Metaverse platforms can interconnect and work harmoniously, fostering a seamless and continuous user experience. The concept of usability will involve an in-depth exploration of the user-friendliness and intuitiveness of Metaverse interfaces, enabling easy navigation and communication.

Finally, the study of Social Influence will provide insight into how social dynamics and peer interactions influence the actions and choices of individuals in the Metaverse. By analyzing these crucial components, we aim to acquire significant perspectives regarding the user's perception of the Metaverse and the determinants contributing to a favourable and engaging virtual encounter.

### 4.1 Platforms interoperability

Metaverse interoperability involves two important things: first, making it easy to use digital elements (like those used to create virtual worlds) in different apps, and second, allowing users to move smoothly between different virtual places without any interruptions, so they have a continuous and connected experience [41]. The crucial factor of platform interoperability significantly influences the users’ perception of the Metaverse. This section combines the findings of many research projects and investigates the relationship between platform interoperability and user perceptions of the Metaverse. Many studies have investigated user perspectives on the Metaverse but with varying degrees of attention on platform interoperability.

For example [15], recognizes the significance of platform interoperability and suggests the creation of contracts to accomplish interoperability across Metaverses without negatively impacting users. Similarly,

[42] emphasizes the importance of resolving interoperability issues to prevent fragmentation of the Metaverse ecosystem and the damaging brand reputation. These results indicate that platform interoperability is crucial in determining how users perceive Metaverse. Table 2 presents some of the studies’ findings of Metaverse Interoperability, while Table 3 shows some of the challenges faced by the Metaverse interoperability.

**Table 2:** Dimensions of the Metaverse interoperability studies.

Aspect	Details	Reference(s)
Narrative Techniques	Importance of user-centric design in the cultural Metaverse.	[43]
Secure Platform Interoperability	Challenges in addressing security and privacy. Open Metaverse with a Zero-Trust Architecture model. Implications on user trust.	[18,44]
Collaboration	Need for standardization. Compatibility fostering cooperation. Influence on user attitudes.	[17]
Overall Impact	Interoperability influences usability, security, and coherence of the Metaverse ecosystem.	[17,22,23,43,45]
Significance	Optimizing user experiences, collaboration, and addressing security through interoperability. Need for more research on its impact on user perspectives.	[15,17,23]

**Table 3:** Interoperability challenges.

Potential Challenges on Metaverse Interoperability Studies	Reference(s)
Interoperability across multiple Metaverses.	[15]
Addressing interoperability uncertainties.	[42]
Establishing unified standards for user credentials.	[42]
Ensuring seamless user experience within the Metaverse.	[22]
Strengthening connections between platforms for better virtual connectivity.	[45]
Addressing security and privacy concerns of extended reality systems.	[44]
Integration of a Zero-Trust Architecture (ZTA) model in an open Metaverse framework.	[18]
Standardization within the Metaverse for coherence and interconnectivity.	[17]

#### 4.1.1 The role of platform interoperability in the adoption of the Metaverse

Platform interoperability remains paramount in defining the trajectory of the Metaverse's growth. Several studies highlight its indispensable nature.

At the heart of the Metaverse's attraction lies its promise of a seamless, integrated experience across diverse platforms [15]. not only digs into this promise but also provides a pathway through creating contracts. These contracts aim to achieve a balance between integration and independent platform functionality, ensuring that users can transition smoothly between virtual spaces without encountering disruptive barriers.

But what makes these transitions meaningful is the content and narrative [43]. underlines the importance of narratives that resonate with the global audience. An interconnected Metaverse will host users from myriad cultures and backgrounds, necessitating a diverse and inclusive content strategy. Crafting universally relatable stories and experiences ensures a sense of belonging, making users more willing to explore further and more profoundly.

Yet, beyond the user narratives, the technical base remains critical. Research from Refs. [22,23] underscores this. Interoperability, while a technological challenge, is also an opportunity. By developing tools, protocols, and services that align with global standards, the Metaverse can evolve into a cohesive ecosystem, broadening its appeal and accessibility.

Within this ecosystem, collaboration stands out as a force multiplier. As highlighted by Ref. [17], when platforms and stakeholders share and collaborate based on common standards, the Metaverse's potential magnifies, making it an irresistible proposition for newcomers.

#### 4.1.2 Platform interoperability and security and privacy concerns

The rise of the Metaverse has brought about a complex world of virtual experiences and interactions that rely a lot on different platforms working together smoothly. Platform interoperability within the Metaverse not only enhances user experience by allowing for seamless movement and interaction across different virtual spaces but also raises significant concerns regarding security and privacy [42]. Interoperability demands a level of openness and shared standards among platforms, which, while fostering a cohesive virtual environment, could potentially expose users to increased risks of data breaches and privacy violations [46].

Security in the Metaverse goes beyond the usual challenges of keeping online spaces safe. The interconnectedness of platforms means that vulnerabilities in one system could have cascading effects throughout the network, jeopardizing user safety and trust [18]. The Metaverse ecosystem, therefore, must address security at both the individual platform level and the collective environment, ensuring that interoperability does not compromise the integrity and confidentiality of user data [23].

Also, privacy worries are bigger in a place where personal and behavioral information is used to tailor the virtual experience. The Metaverse's capacity to offer personalized virtual experiences relies on sensitive user data, which must be safeguarded against misuse. As platforms interoperate, the management of such data becomes complex, necessitating robust privacy governance frameworks that transcend single-platform policies [47].

Confronting these concerns, innovative solutions such as blockchain technology are being explored for their potential to secure transactions and authenticate user identities across the Metaverse [16]. Blockchain's decentralized nature and encryption capabilities present a compelling case for securing user data and establishing trust without hampering the interoperability of virtual spaces [17].

The overarching aim is to strike a balance between creating an integrated, user-friendly Metaverse and maintaining a fortress of security and privacy. This goal calls for collaborative efforts among technologists, policymakers, and the Metaverse community to establish and enforce standards that prioritize user safety, data protection, and ethical usage of technology [12]. As the Metaverse continues to evolve, the strategies adopted

to resolve these challenges will be pivotal in shaping its trajectory and the user's trust in these burgeoning digital worlds.

#### 4.1.3 Challenges hurdling platform interoperability

Even as the potential of the Metaverse soars, challenges cast shadows on the path to seamless interoperability. Foremost among these challenges is ensuring user security and privacy [44]. provides a true reflection of the current situation. As the virtual boundaries blur, ensuring these interconnected realms remain safe from malicious threats is paramount. Every breach and exploitation threatens individual users and the credibility

of the entire Metaverse.

To tackle these threats, innovative solutions emerge [18]. showcases the promise of the Zero-Trust Architecture (ZTA) model. But its adoption isn't straightforward. Merging security models like ZTA with the inherent openness that interoperability demands is a Herculean task that requires rethinking traditional security paradigms.

Coupled with security is the challenge of identity and reputation. As highlighted by Ref. [42], in a vast universe of platforms, ensuring that users can retain their identities and that brands can uphold their reputations is complex. The solution lies in creating unified standards. However, this again presents a challenge: achieving a consensus in a realm as diverse and multifaceted as the Metaverse. Balancing individual platform aspirations with collective goals requires diplomacy, foresight, and a commitment to a shared vision.

With the potential to reshape our virtual interactions and the challenges that threaten its realization, the Metaverse's journey is an epic saga in the making. Its promises and perils will shape the future of digital interactions, demanding attention, innovation, and collaboration from stakeholders worldwide.

### 4.2 Usability

The Metaverse—immersive digital environments—has emerged due to fast technological innovation. This virtual reality arena lets users enjoy socializing, entertainment, education, and business. As the Metaverse evolves, usability and user experience become vital to its success and widespread acceptance.

The ability of users to effectively participate and traverse Metaverse ecosystems is significantly impacted by the usability of systems, which refers to their efficiency and ease of use. A superb user experience is contingent upon essential elements such as intuitive interfaces, seamless interactions, and engaging content. Hence, it is imperative to identify and rectify usability concerns to ensure that the Metaverse provides users with an accessible, immersive, and gratifying milieu. Table 4 summarises the different aspects of the Metaverse usability. The table categorizes these issues into several key areas: General Usability, AR Usability, User Experience in Metaverse Contexts, Ethical and Social Implications, Usability Challenges and Limitations, Augmented Reality Experiences, Usability of Metaverse in Healthcare, Metaverse Technology, and Ethical Considerations. Each category addresses challenges such as user experience, ergonomic concerns, data privacy, health and environmental impacts, security, content generation, AI transparency, ethical and social concerns like cyberbullying and racism, and technical limitations like accessibility and ease of use. Furthermore, Table 5 highlights some of the important findings of the Metaverse usability studies.

**Table 4:** Aspects of Metaverse usability research.

Aspect of Usability	Challenges/Concerns	Reference(s)
General Usability	Context immersion, User Experience (UX)	[43]
	Enhancing user interface, data visualization, ergonomic concerns	[9]
	Transaction and participation costs, Data privacy and ethics, Health concerns, Environmental impact, Shifting preferences	[20]
AR Usability	Dark side of virtual ecosystem, Ethical concerns	[19]
	Pragmatic and Hedonic qualities, Error counting	[48]
User Experience in Metaverse Contexts	Security and Privacy, Content Generation, AI Predictions, Transparency of AI systems	[35]
	Incorporation of external data, Patient-centric healthcare experiences	[7]
	Self-efficiency, Social norm, Perceived curiosity, Perceived pleasure, Price considerations	[10]
Ethical and Social Implications	Security and Privacy concerns, Customer profiling, Aggressive marketing, Cyberbullying, Racism, Digital personality mining, Digital harassment	[16]
	Privacy and security issues, Data privacy and cybersecurity regulations, Disparities in access	[25]
Usability Challenges and Limitations	Access, Ease-of-use, Lack of developed ecosystem	[18]
	Adoption and outcomes, Technological standards, Accessibility for older individuals, Comparison to in-person visits	[23]
Augmented Reality Experiences	User-friendly designs in educational and training contexts	[19]
Usability of Metaverse in Healthcare	Usability impacted by the delivery of patient-centric healthcare experiences	[7]
Metaverse Technology	Relationship between usability and user acceptance	[10]
Ethical Considerations	Ethical responsibility, Security, Privacy, Digital harassment	[16]
	Undemocratic and unethical practices due to surveillance capitalism	[47]

**Table 5:** Sample findings of studies of Usability in the Metaverse.

Reference	Findings Summary
[43]	Context Immersion (The article mentions a study by Kim that discusses immersion in augmented reality (AR) as context immersion. This refers to how AR logically interacts with [43] the user's real physical environment, incorporating dimensions such as time and location-based context, object-based context, and user-based context.) User Experience (UX) (The article emphasizes the importance of UX in immersive environments and the need for research to understand and optimize interactive communication content for a better user experience)
[9]	enhancing the user interface, data visualization, and considering ergonomic considerations Transaction and participation costs. Data privacy and ethics concerns. Health-related concerns, Environmental impact. Shifting preferences (The demand for the Metaverse's products, services, and way of living and working is influenced by shifting preferences)
[20]	
[19]	challenges, risks, and ethical considerations associated with the use and implementation of the metaverse include: the potential dark side of the virtual ecosystem, to which AR serves as a gateway, virtual ecosystems, including the metaverse, and highlights the importance of research on these ecosystems, the technologies used to access them (such as AR), and the ethical concerns that arise
[48]	Pragmatic qualities. Hedonic qualities. Dual-task exercise and error counting. Potential of the Metaverse (The article highlights the potential of the Metaverse in connecting people together regardless of their location, which reinforces its usability).
[35]	Security and Privacy. Content Generation. AI Model Predictions. Transparency of AI Systems.
[7]	the incorporation of external data and the delivery of patient-centric healthcare experiences impact the usability of metaverse services in the healthcare industry
[10]	Self-efficiency. Social norm. Perceived curiosity. Perceived pleasure. Price
[16]	Security and Privacy Concerns. Customer Profiling. Aggressive Marketing. Cyberbullying and Racism.Digital Personality Mining. Digital Harassment.
[25]	Privacy and Security Issues. Data Privacy and Cybersecurity Regulations. Disparities in Access. Access. Ease-of-Use. Lack of Developed Ecosystem.
[18]	
[23]	Adoption and outcomes.Technological standards and protocols.Accessibility for older individuals. Comparison to in-person visits.

#### 4.2.1 Usability in Metaverse ecosystems

Several studies have investigated the efficacy of Metaverse ecosystems [15]. briefly mentions the possibility of stakeholders co-creating value and the need for enhanced usability and user experience [43]. investigates the efficacy and user experience of the cultural Metaverse, emphasizing the significance of user-friendly interfaces, informative content, and engaging interactions. In Ref. [9], the authors address usability explicitly and propose enhancements, such as enhancing the user interface, data visualization, and ergonomic considerations. While.

[20] discusses usability, emphasizing its broader implications for the design and functionality of the Metaverse. According to Ref. [49], the usability of the metaverse is contingent upon three foundational elements: robust infrastructure, encompassing powerful computing capabilities and high-speed, dependable internet; user-friendly design; and content that is both engaging and pertinent to users. The significance of compelling content persists, notwithstanding the user-friendliness of the application.

#### 4.2.2 Usability and user experience

Numerous studies have dig into the effectiveness and user experience within various Metaverse scenarios. For instance Ref. [43], explores the cultural aspects of the Metaverse, highlighting how usability plays a pivotal role in shaping user experiences. This research underscores the importance of intuitive design in creating engaging virtual environments.

In a similar vein [19], underscores the criticality of user-friendly and intuitive designs, particularly in educational and training settings. This study suggests that ease of use is fundamental in facilitating effective learning and skill acquisition in virtual spaces.

[48] shifts the focus to the realm of augmented reality, showcasing the enhanced user experiences achieved through the Digital Storytelling in the Environment (DSTE) framework. This research demonstrates how usability in augmented reality can significantly enrich user engagement and interaction.

Further expanding on this theme [35], examines the role of usability in relation to avatars and haptic feedback within 3D virtual environments. This study emphasizes the importance of sophisticated avatar design and advanced haptic technologies in boosting user immersion and satisfaction.

Lastly [7], addresses the usability of Metaverse services, particularly spotlighting the integration of external data and the creation of patient-centric healthcare experiences. This research highlights the Metaverse's potential to revolutionize healthcare delivery through personalized and interactive virtual services.

#### 4.2.3 Usability's ethical and social implications

Many studies have addressed the ethical and social implications that can influence the Metaverse's efficacy [16]. prioritizes ethical responsibility and sustainability in the Metaverse business model, addressing security, privacy, and digital harassment concerns. While [50], focuses on information ethics while implicitly recognizing the importance of usability in designing and implementing emergent technologies that benefit all users. Moreover,

[47] emphasizes the potential undemocratic and unethical practices deriving from surveillance capitalism, which could impact Metaverse user experiences.

#### 4.2.4 Usability and security and privacy concerns

The usability of the Metaverse, a key factor in its adoption and user satisfaction, intertwines closely with security and privacy considerations. As users navigate through virtual worlds, the ease of interaction with various platforms significantly influences their overall experience. However, this seamless navigation raises security and privacy issues as the protection of user data becomes increasingly challenging [23].

Usability, defined by the intuitive design and user-friendly interfaces of Metaverse platforms, ensures that individuals can engage with virtual environments effectively. Yet, the complexity of creating such environments, which are both accessible and secure, cannot be understated. As [18] points out, the Metaverse architecture necessitates robust security measures to safeguard against potential cyber threats, which may exploit usability features to gain unauthorized access to personal and sensitive data.

Furthermore, privacy concerns in the Metaverse are heightened due to the reliance on personal and behavioral data to customize user experiences [47]. This data, while enhancing usability by tailoring experiences to individual preferences, poses significant risks if not adequately protected. Users’ digital footprints can reveal much about their habits and preferences, making privacy a paramount concern.

To address these challenges [16], suggests the implementation of blockchain technology as a means to enhance security and privacy in the Metaverse. Blockchain's decentralized nature offers a potential solution for maintaining user privacy while ensuring the interoperability of different platforms. This technology, by providing a secure and transparent way to handle data transactions, could mitigate some of the inherent tensions between usability and privacy concerns.

In summary, while usability is crucial for ensuring that the Metaverse is accessible and engaging for users, it must not be pursued at the expense of security and privacy. Balancing these aspects requires innovative solutions and ongoing vigilance to protect users in these vast virtual landscapes. As the Metaverse continues to evolve, the development of technologies and frameworks that support both usability and user protection will be critical for its sustainable growth.

#### 4.2.5 Technology empowerment in the Metaverse for individuals with disabilities

The rapid development of the Metaverse has prompted conversations on fair and inclusive availability, specifically for individuals with disabilities [51]. dig into this, questioning the Metaverse's current capacity for inclusivity and exploring necessary enhancements for equal participation. Their work underscores the importance of consulting those with disabilities to understand their Metaverse experiences and preferences, which should inform the development of tailored design solutions that are further refined by hands-on virtual reality (VR) and augmented reality (AR) experiments.

[52] contribute to this field by presenting an innovative educational methodology within a 3D virtual reality framework, targeting students with disabilities. Their intelligent system marries the pedagogical strengths of Moodle, the simulation prowess of Sloodle, and the virtual environment management of OpenSim, culminating in a meta-learning platform that has garnered affirmative responses for its rapid, impactful, and captivating educational experiences.

Parallel to these educational advancements [53], advocates for the active involvement of physically disabled artists in shaping the Metaverse. The proposed framework emphasizes the criticality of their contribution to decentralizing these platforms, ensuring disabled creatives are not mere consumers but also key players in content creation. It calls for necessary infrastructural adjustments and regulatory actions to secure their effective participation in Metaverse initiatives.

[54] introduces a pioneering vocational training program within the Metaverse, crafted to support adults with disabilities. Amidst the challenges of the Fourth Industrial Revolution and the aftermath of the COVID-19 pandemic, this program has demonstrated success in enhancing participants’ social skills, problem-solving in workplace settings, and proficiency in online interviews. Its significance is anchored in its forward-thinking approach, adapting vocational interventions to the digital dimensions of the Metaverse, which transcends traditional education and training models.

#### 4.2.6 Users’ acceptance of the Metaverse and Usability Challenges and Limitations

The burgeoning field of Metaverse research has increasingly focused on the interplay between usability, user acceptability, and the overall user experience within these virtual environments. A synthesis of recent studies reveals a multifaceted examination of the challenges and opportunities inherent in Metaverse technology.

[25] underscores the critical importance of accessibility and inclusivity in the Metaverse, highlighting barriers related to infrastructure access, atmospheric simulation, and tactile stimuli. This perspective is crucial in understanding the broader implications of Metaverse applicability and its potential limitations. Similarly,

[18] digs into the physical usability issues posed by the current generation of headgear, emphasizing the need for ergonomic improvements in headset design to enhance user comfort and usability.

Complementing these insights [23], explores the user perception challenges in adopting Metaverse technologies, particularly in healthcare settings. This study indicates how user perceptions can indirectly influence the efficacy and acceptance of these technologies, thereby impacting their overall success.

In a parallel vein [10], investigates the effectiveness of Metaverse technology, focusing on its impact on user acceptability and perceptions. This research is pivotal in understanding how the functionality of Metaverse environments influences user engagement and satisfaction [55].contributes to this discourse by offering guidelines for designing intuitive and useable Metaverse spaces, thereby underscoring the importance.

Of user-centered design principles. Furthermore [21], emphasizes the significance of usability and user interface design in virtual worlds, advocating for the development of user-friendly interfaces to enhance interaction and user experience.

Collectively, these studies highlight the critical importance of usability within the Metaverse ecosystem. They underscore the need for creating user-friendly interfaces, ensuring informative and interactive content, and incorporating user feedback to enhance usability. The challenges and limitations identified across these studies emphasize the necessity of addressing issues related to accessibility, comfort, and equitable access within the Metaverse. This body of research collectively suggests that the success and widespread adoption of Metaverse technologies hinge on a concerted effort to resolve these usability concerns, thereby ensuring a positive and inclusive user experience.

### 4.3 Social influence

The advent of the Metaverse has sparked significant scholarly attention, leading to a proliferation of research exploring the various dimensions of this virtual realm. A key focus among these studies is the impact of social influence within the Metaverse, digging into how it shapes user behavior, decision-making processes, and the overall user experience.

This section explores a selection of studies from our comprehensive literature review. These investigations shed light on social influence in the Metaverse and the range of outcomes it produces. This exploration enhances our understanding of social dynamics in virtual environments and offers valuable insights into the broader implications of these interactions for the evolving digital landscape.

Table 6 presents a comprehensive summary of key findings on social influence within the Metaverse, categorized into distinct areas. It highlights factors influencing Metaverse usage, such as ethical concerns, technological literacy, and trust in virtual environments [42,56]. The role of social engineering and the impact of social norms and peer pressure are also examined. Additionally, the table explores the intersection of augmented reality (AR), virtual reality (VR), and social media, discussing their implications on collaboration, healthcare, and consumer behavior, with insights from Refs. [19,57]. Finally, it addresses the broader benefits and challenges of the Metaverse, including its applications in healthcare, governance, and education, as well as concerns about misinformation and social media risks [12,17]. This synthesis provides a comprehensive understanding of the effects of social influence in the evolving landscape of the Metaverse.

**Table 6:** Summary of findings on social influence in the Metaverse.

Category	Finding	Reference
Factors Influencing Metaverse Usage	Ethical and governance issues, technological literacy, self-efficacy, consumer trust in virtual settings, and role of technology familiarity	[15,31,42,56]
Social Influence	Deceptive behaviors, manipulation, social engineering, cyberbullying, and the role of social norms and peer pressure	[25,42,56]
Role of AR, VR, and Social Media	Augmented reality's collaboration potential, anthropomorphism in healthcare, cyberbullying in social media, and social influence in AR	[19,35,48,57]
Consumer Behavior	Social media's role in purchases, threats in VR, VR in healthcare adoption, and manipulative algorithms in purchases	[9,15,44,58]
Metaverse Benefits and Challenges	Advantages in healthcare, governance importance, misinformation concerns, social media risks, and educational roles	[12,14,17,31,47]

#### 4.3.1 Social influence in the Metaverse

Several studies discuss social influence in the context of the Metaverse [42]. discusses the potential for avatars controlled by malicious human users to engage in deceptive and evil behavior, indicating social influence through.

impersonation, manipulation, and social engineering [56]. employs social cognitive theory to understand how individuals perceive and evaluate behaviours related to the Metaverse, emphasizing the role of social influence from peers, family, and social networks. This study analyses the emotional contagion effect and negative social impacts, such as cyberbullying and prejudice that must be addressed to develop a sustainable and ethical Metaverse. Moreover [25], emphasizes the role of social influence in influencing consumer behavior and decision-making in virtual environments, focusing on the impact of social norms and peer pressure in the Metaverse. However [59], finds that students in higher education may not be influenced by others' experiences with educational Metaverse platforms. As students become more familiar with Metaverse tech, they're less likely to be influenced by support from their social circles, which is against previous research conclusions.

[19] discusses how augmented reality (AR) can facilitate collaboration, creativity, and user engagement and implies that social influence may play a role in these processes. In Ref. [57], the authors examine the impact of anthropomorphism and affective receptivity on individuals’ intent to use digitally-based healthcare services; however, social influence is not explicitly addressed [35]. examines cyberbullying and cyberaggression in social media, which are forms of social influence [48]. evaluates modalities of information presentation in a virtual environment, considering the potential impact of social influence in collaborative or communicative augmented reality applications.

#### 4.3.2 Role of social influence on the adoption of Metaverse

The Metaverse, often envisaged as the next frontier in digital evolution, is fundamentally driven by its social components. However, its adoption isn't merely dictated by technology; the underlying currents of social influence play a formidable role. Understanding how individuals and communities perceive, accept, or resist the Metaverse requires digging into the intricacies of social influence.

One of the primary facilitators of Metaverse adoption is the element of trust and familiarity. As elucidated by Ref. [15], trust in the virtual realm is paramount. This trust extends beyond system reliability and encompasses trust in virtual identities, interactions, and the overall sense of community [31]. emphasizes that individuals familiar with digital technologies, especially immersive ones, are more predisposed to embracing the Metaverse, bridging the often-talked-about digital divide.

Another salient component is peer influence. Humans, by nature, are influenced by their immediate social circles. Whether fashion, language, or technology adoption, we often look to our peers, consciously or subconsciously, to guide our decisions [56]. posits that the Metaverse, despite its vast and boundless nature, isn't exempt from this fundamental human trait. The recommendations, experiences, and narratives shared within our social networks are pivotal in shaping our perspectives about the Metaverse.

Lastly, social norms and behaviors underpin all interactions in the Metaverse. Just as in the physical world, the virtual domain of the Metaverse has its norms and unspoken rules. These guidelines, as discussed by Refs. [10,25], determine user behaviour, how interactions occur, how conflicts are resolved, and how communities are formed and sustained.

#### 4.3.3 Social influence and user trust

In the context of the Metaverse, social influence emerges as a pivotal factor shaping user trust. The dynamics of social interactions within these virtual environments significantly impact users' perceptions and their confidence in the platform's security and privacy measures [15]. As users navigate through these digital realms, the endorsements or criticisms from their social networks can significantly influence their level of trust in the Metaverse's infrastructure and its capacity to safeguard personal information [42].

The role of social influence in the Metaverse extends beyond mere peer interactions; it encompasses the broader societal norms and cultural expectations that users bring into these virtual spaces. Such social norms can dictate the acceptable boundaries of behavior within the Metaverse, further influencing user trust. When users observe that these norms are upheld, their trust in the platform is reinforced. Conversely, witnessing or experiencing breaches of these social contracts can erode trust rapidly [56].

Furthermore, the phenomenon of social influence within the Metaverse is intricately linked to the platform's usability and the design of user experiences. Platforms that facilitate positive social interactions and provide users with controls to manage their privacy are more likely to engender trust [18]. On the other hand, platforms that fail to address users' social and privacy concerns may foster a climate of mistrust and apprehension [23]. Trust in the Metaverse is not solely determined by the platform's technical security measures; it is also shaped by the quality of social interactions that it enables. Users place their trust in platforms where they feel their social expectations are met and their personal boundaries respected. Hence, addressing social influence is crucial for building and maintaining user trust in the Metaverse. Creating environments that support positive social interactions, respect user privacy, and reflect the social norms of their user base is essential for fostering

A trusted and secure Metaverse [16].

In summary, the interplay between social influence and user trust underscores the need for Metaverse platforms to not only focus on robust security and privacy mechanisms but also on cultivating a socially positive and respectful virtual environment. This dual focus is crucial for engendering a sense of trust among users, ensuring their continued engagement and participation in the Metaverse.

#### 4.3.4 Psychological and social effects of Metaverse

Prolonged interaction within the Metaverse can elicit a spectrum of psychological responses, ranging from enhanced feelings of connectedness and community to potential risks of isolation and disassociation from the physical world [15]. The immersive nature of the Metaverse, while offering novel avenues for socialization and engagement, raises pertinent questions regarding its long-term effects on mental health and social behaviors. Continuous exposure to virtual interactions could potentially alter users' perceptions of social norms and expectations, leading to an adjustment in how relationships are valued and maintained in the real world [42]. Moreover, the blurring lines between virtual and physical identities could prompt introspection about self-concept and social identity, affecting individuals’ psychological well-being [56].

The link between pulling away from social activities and getting deeply involved in the Metaverse requires careful attention. As users invest more time in virtual worlds, the propensity to retreat from physical interactions could intensify, potentially leading to a form of social isolation that impacts their real-world social networks and support systems [18]. This shift may influence individual psychological health and affect the broader social fabric, as the nature of community and belonging transforms [23].

The potential for the Metaverse to serve as a double-edged sword in terms of psychological and social health underscores the need for comprehensive research into its long-term impacts. While the virtual worlds offer escape, entertainment, and new modalities of connection, it is critical to understand how these benefits balance with the risks of decreased physical world engagement and its subsequent effects on mental health and social wellbeing [16].

#### 4.3.5 Impact of social media and virtual reality on consumer behavior

Various studies investigate the influence of social media and virtual reality on consumer behavior [15]. examines the impact of social media marketing features on consumer purchase decisions in the fast-food industry, focusing on the mediating role of brand trust [44]. emphasizes the potential of virtual reality for social interactions and self-expression and the possibility of social engineering assaults in virtual reality environments.

In [9], the authors examine the influence of individual motivation and social influence on telemedicine adoption and conclude that social influence can positively influence the public's behavioral intention to adopt VR-based telerehabilitation technology. Moreover [58], examines the use of manipulative social algorithms and their impact on users' purchasing decisions on conversational commerce platforms.

### 4.4 Challenges that impact the role of social influence in the adoption of Metaverse

The bridge between the Metaverse and its potential adopters isn't without its hurdles, many of which are socially rooted. Foremost are the ethical concerns. The potential for deceptive behaviors, impersonation, or even more sinister forms of manipulation raises alarms [42]. underscores the gravity of these concerns, suggesting that unchecked trust could undermine the foundation of trust essential for the Metaverse's growth. Coupled with ethical considerations is the risk of cyberbullying. As virtual realms offer anonymity and detachment from real-world consequences, they sometimes become a place for negative influences. The Metaverse needs robust safeguards against threats like cyberbullying to ensure that it remains a space of positive engagement, as pointed out by Refs. [35,56].

Another challenge is misinformation. In an era where fake news can have real-world consequences, the Metaverse isn't immune, as highlighted by Ref. [14]. Misinformation, especially when driven by vested interests, can skew perceptions and impede genuine social interactions.

Governance in the Metaverse also presents a unique set of challenges. As [17,47] explain, ensuring democratic, transparent, and equitable governance within the Metaverse isn't just an ideal—it's a necessity. Without it, the metaverse risks becoming a small-group-controlled domain, which would undermine its potential as a place for everyone.

Lastly, the influence of manipulative algorithms poses a significant challenge. Algorithms can shape narratives, influence decisions, and even manipulate behaviors. Ensuring that these algorithms act as facilitators rather than controllers is crucial, a sentiment echoed by Ref. [58] (Table 7 summarises the challenges impacting social influence in the Metaverse).

**Table 7:** Challenges impacting social influence in the Metaverse.

Challenge	Description	Reference
Ethical Issues	Concerns around deceptive behaviours, impersonation, and unscrupulous activities by avatars controlled by malicious users.	[42]
Cyberbullying	Presence of negative influences in the Metaverse, such as cyberbullying, can affect user behaviour and experiences.	[35,56]
Misinformation	The potential for misinformation, agenda-driven corporate media influence, and mass formation hysteria.	[14]
Governance Issues	Risks associated with undemocratic and unscrupulous governance practices within the Metaverse, emphasizing the need for robust governance.	[17,47]
Manipulative Algorithms	The use of manipulative social algorithms that can unduly influence user decisions, especially in commerce platforms.	[58]

#### 4.4.1 Social influence and user acceptance

Several studies investigate the relationship between user acceptability of Metaverse technology and social influence [10]. examines the extended technology acceptance model and identifies subjective norm related to social influence as a factor influencing consumers’ adoption of Metaverse technology [55]. also employs the UTAUT model and finds that social influence substantially impacts usage intention, purchase intention, and word-of-mouth intention, as well as increasing satisfaction with the Metaverse platform.

Moreover [26], examines the potential influence of the Metaverse on social influence, focusing on the creation of immersive experiences that can influence consumer behaviour and the promotion of social interaction and community development.

These studies provide significant insights into the complex and diverse aspects of social influence in the Metaverse and its related technologies. They emphasize the noteworthy influence of social factors on user behaviour, cognitive functions, and overall interactions within the digital domain. By comprehensively comprehending and strategically handling the consequences of social influence, relevant parties can strive to establish a durable and morally sound Metaverse.

## 5 Discussion

### 5.1 Practical implications

The Metaverse stands at the precipice of transforming digital interaction and the fabric of our societal, educational, and healthcare frameworks, promising an inclusive future that transcends cultural, age-related, and socio-economic boundaries. Drawing from the survey's insights, we dig deeper into how the principles of interoperability, usability, and social influence shape these transformations, emphasizing the imperative of inclusivity and diversity in the Metaverse's evolution [7,10,15,19,20,23,43].

The Metaverse's potential in healthcare is monumental, promising a future where quality care and advanced medical training are universally accessible. Through the seamless integration of VR and AR technologies, healthcare professionals can simulate complex medical procedures, offering a hands-on learning experience without the constraints of geography or resource availability. This democratization of medical knowledge and skills is crucial for bridging the global healthcare divide, ensuring that professionals, irrespective of their socio-economic or cultural backgrounds, have the same high-quality training and resources. Thus, the Metaverse is a hope for universally fair healthcare delivery, enabling inclusive and inventive patient care [7,19]. In education, the Metaverse's role is transformative, offering immersive learning experiences that captivate students' imagination and foster a deeper understanding of complex concepts. The key to unlocking this potential lies in the usability of educational platforms within the Metaverse. By ensuring these platforms are intuitive and accessible, learners from various cultural backgrounds and age groups can benefit equally from the Metaverse's educational offerings. Moreover, the Metaverse's inherent capacity to support dynamic social interactions among students can significantly enhance collaborative learning, making education a more

Engaging, effective, and inclusive. In this way, the Metaverse promises to overcome traditional educational barriers, providing equitable learning opportunities for all [15,43].

The Metaverse's implications for business extend far beyond technology, heralding a new era of economic inclusion and opportunity. By creating a virtual marketplace without physical or geographical barriers, the Metaverse offers unprecedented access to global markets, especially for small businesses and entrepreneurs from underrepresented regions. This level of access is pivotal for stimulating economic growth and fostering diversity within the global marketplace. Furthermore, the Metaverse's support for remote work and collaboration can significantly broaden employment opportunities, offering a lifeline to individuals in remote or economically disadvantaged areas. The Metaverse can serve as a powerful equalizer in the global economy, promoting inclusivity and diversity in business practices and opportunities [10].

As the Metaverse grows, so does its environmental footprint, making sustainable development an imperative aspect of its expansion. By prioritizing developing and adopting energy-efficient technologies and advocating for sustainable virtual world designs, the Metaverse community can ensure that this digital evolution does not come at an environmental cost. This commitment to sustainability is not just about preserving our planet for future generations; it's also about ensuring that the Metaverse is a space that respects and reflects the diverse values and priorities of its global user base, including a shared concern for environmental stewardship [20].

The vision of the Metaverse as an inclusive digital society where every user, regardless of physical ability, cultural background, or economic situation, can explore, interact, and contribute equally is ambitious and necessary. Achieving this vision requires a concerted effort to ensure interoperability across diverse platforms, enhance the usability of virtual environments, and cultivate positive social influences within these spaces. By addressing these challenges with an unwavering commitment to inclusivity and diversity, the Metaverse can become a digital interaction model that mirrors and enhances the richness and complexity of our physical world [23].

In sum, the journey toward a fully realized Metaverse is fraught with challenges but also brimming with potential. By embracing the principles of interoperability, usability, and social influence and by foregrounding the values of inclusivity and diversity, we can steer the development of the Metaverse toward a future where digital and physical realities coalesce, offering unparalleled opportunities for learning, healing, working, and thriving together, across all divides [7,10,15,19,20,23,43].

### 5.2 Theoretical implications

The theoretical landscape of the Metaverse is as vast and varied as the virtual worlds it comprises. At its core, the Metaverse challenges traditional paradigms of user experience and usability, calling for the creation of new theoretical frameworks that can effectively capture the complex nature of human interaction in these immersive environments. The Metaverse's unique blend of interactivity and immersion demands a reevaluation of existing models of user experience, pushing scholars toT deeply and carefully analyze the aspects of involvement, usability, and functionality in virtual environments [9,43]. This evolution in understanding user experience within digital realms opens up avenues for significant advancements in how we conceptualize and design for user interactions in virtual environments.

Simultaneously, the social structure of the Metaverse provides a complex and diverse subject for theoretical investigation, particularly in the realm of digital socialization. Virtual communities’ influence, the development of social norms in these digital spaces, and peer pressure all have a familiar and novel impact on user behavior. This dynamic presents an opportunity to expand existing theories of social influence, applying them to the unique context of the Metaverse to uncover how virtual interactions influence human behavior, decision-making processes, and social dynamics at large [42,56]. Such insights not only contribute to our understanding of digital socialization but also highlight the potential of the Metaverse to redefine social interactions in the digital age.

Moreover, the necessity for ethical and governance frameworks within the Metaverse underscores the theoretical implications for digital ethics and virtual governance. As the Metaverse continues to grow, developing these frameworks becomes imperative to ensure that innovation does not outpace considerations for user rights, privacy, and ethical conduct. Theoretical exploration in these domains can guide the formulation of policies and guidelines that strive for a balance between innovation and ethical responsibility, informing the governance of virtual worlds in a manner that respects user autonomy and promotes fairness [17,47].

Additionally, the Metaverse prompts a reexamination of the psychological and societal impacts of prolonged immersion in virtual environments. The intersection of digital sociology, psychology, and anthropology with the study of the Metaverse can yield new understandings of how virtual experiences affect human behavior, societal norms, and interpersonal relationships. This exploration is crucial in predicting the long-term effects of the Metaverse on society and individual well-being, paving the way for a digital future that is mindful of its impact on human psychology and social structures [35,48].

Finally, the economic structure of the Metaverse presents new theoretical difficulties, namely, with the intersection of virtual economies with real-world financial systems. Understanding the dynamics of virtual economies, monetization strategies, and the role of technologies such as blockchain is essential for comprehending

the economic implications of the Metaverse. This theoretical exploration can provide insights into the economic viability of virtual worlds, their impact on global economic models, and the opportunities and challenges they present for economic participation and growth [10,15].

In essence, the Metaverse, with its rapidly evolving nature, presents a multifaceted array of theoretical and practical implications. Navigating these implications requires a multidisciplinary approach that melds insights from technology, sociology, psychology, ethics, and economics. By embracing this comprehensive perspective, we can harness the full potential of the Metaverse to create virtual experiences that are not only technologically advanced but also socially responsible, economically viable, and inclusive, laying the groundwork for a future that enriches both digital and physical realities.

## 6 Future research directions

Based on the extensive review of the Metaverse, several critical areas for future research emerge. These areas encompass its technological advancements, user experience, usability, social influence, and ethical considerations. These areas address the current gaps and anticipate the Metaverse's evolving nature.

One significant area for future research is the enhanced integration of emerging technologies. This includes exploring integrating newer technologies within the Metaverse, like quantum computing, advanced AI algorithms, and next-generation VR and AR systems. Investigating the implications of these technologies on user experience, data processing capabilities, and the overall functionality of the Metaverse is crucial for its advancement [15,43].

Another vital area is the optimization of user experience in the Metaverse. There is a need for in-depth studies to optimize the user experience, including research on intuitive interface design, user engagement strategies, and personalized content delivery. Particular attention should be given to the usability of the Metaverse in specialized fields like healthcare and education [7,19].

Data privacy and security in the Metaverse also present a significant area for future research. As the Metaverse continues to evolve, the issues of data privacy and security become increasingly complex. Future research should focus on developing robust encryption methods, secure data storage solutions, and privacy-preserving techniques tailored for the Metaverse environment [16,25].

Ethical and governance frameworks in the Metaverse are another critical area for research. Comprehensive research on ethical frameworks and governance models suitable for the Metaverse is needed. This includes studies on regulatory policies, ethical guidelines for AI and VR content, and frameworks to ensure equitable access and prevent digital discrimination [17,47].

The impact of social influence within virtual environments is another area requiring further research. Understanding the dynamics of social influence within the Metaverse, including the effects of virtual communities, social norms, and peer pressure on user behavior is essential. This also encompasses the study of cyberbullying, misinformation, and the role of social media within these virtual spaces [42,56].

Sustainable development and the environmental impact of the Metaverse are increasingly important. As the Metaverse grows, its environmental impact becomes an important study area. Future research should focus on sustainable development practices in the Metaverse, including energy-efficient computing technologies and eco-friendly virtual environment designs [20].

The long-term psychological and societal impacts of the Metaverse are still largely unexplored and present a rich area for future research. Investigating the psychological impacts of prolonged exposure to virtual environments, changes in social interactions, and the potential for the Metaverse to influence real-world behaviors and societal norms is crucial [35,48].

Accessibility and inclusivity in the Metaverse are also critical areas for future research. Making the Metaverse accessible to diverse populations, including those with disabilities and individuals from various socio-economic backgrounds, is essential. This includes the development of adaptive technologies and inclusive design principles [18,23].

Economic models and monetization strategies within the Metaverse also present a rich area for research. Exploring sustainable economic models and the impact of virtual economies on real-world financial systems is crucial for understanding the economic aspects of the Metaverse, including virtual economies, monetization strategies, and blockchain integration [10,15].

Finally, considering the Metaverse from a global perspective is essential. Research should explore how different cultures interact within virtual spaces, the impact of cross-cultural exchanges, and the development of culturally sensitive content and interactions. This global perspective is crucial for understanding the Metaverse's impact on a worldwide scale [26,55].

These areas of future research not only address the current challenges within the Metaverse but also pave the way for its responsible and sustainable growth. As the Metaverse continues to evolve, these research areas will be crucial in shaping its trajectory and ensuring its positive impact on society and individuals.

## 7 Conclusion and research limitations

As we conclude this extensive exploration of the Metaverse, it is imperative to integrate an understanding of the inherent research limitations that frame our current comprehension of this multifaceted and rapidly evolving digital realm. These limitations are not just boundaries but also signposts, directing us toward areas ripe for future investigation and providing context for the insights we have gleaned.

The Metaverse, characterized by its dynamic nature and underpinned by technologies like VR, AR, AI, and blockchain, is in a state of constant flux. This ever-evolving landscape poses a significant challenge: the findings and understandings we develop today may quickly become outdated as new technologies and applications emerge. Continuous research and adaptation are essential to keep abreast of these rapid advancements and to maintain a relevant and accurate understanding of the Metaverse.

A notable limitation in the current body of research is its predominantly exploratory and theoretical nature. The Metaverse, being in its early stages, lacks a robust pool of empirical data and longitudinal studies. This gap limits our ability to draw definitive, long-term conclusions about its impacts and future developments. As the Metaverse matures and more data becomes available, there will be opportunities to validate, refine, and possibly reframe these initial findings.

Furthermore, there is a tendency in existing research to concentrate heavily on the technological facets of the Metaverse, often at the expense of exploring its social, psychological, and ethical dimensions. While technological innovation is a key driver, understanding the human aspect of interactions within these virtual spaces is equally critical. Future research should strive for a more balanced approach, one that integrates the technological with the humanistic aspects of the Metaverse experience.

Another critical limitation is the geographical and demographic skewness of current research. Much of the existing literature reflects perspectives from technologically advanced regions, potentially neglecting the diverse global viewpoints and the realities of the digital divide. Future studies should aim to be more inclusive, taking into account the varying levels of technological access and literacy across different global regions and socio-economic groups.

Lastly, the ethical and governance frameworks within the Metaverse are still developing. The absence of comprehensive and established standards and regulations makes it challenging to fully assess and predict the long-term societal implications of the Metaverse. As these frameworks evolve, they will undoubtedly provide a solid foundation for future research endeavors.

While the current research offers valuable insights into the Metaverse, acknowledging these limitations underscores the need for ongoing, diverse, and comprehensive research efforts. Such endeavors will not only deepen our understanding of the Metaverse but will also guide its evolution towards a more inclusive, ethical, and user-centric future.

## Data availability

Data will be made available on request.

## CRediT authorship contribution statement

**Mousa Al-kfairy:** Writing – review & editing, Writing – original draft, Visualization, Validation, Supervision, Software, Resources, Project administration, Methodology, Investigation, Funding acquisition, Formal analysis, Data curation, Conceptualization. **Ayham Alomari:** Writing – review & editing, Writing – original draft, Visualization, Validation, Supervision, Software, Resources, Methodology, Investigation, Formal analysis, Data curation, Conceptualization. **Mahmood Al-Bashayreh:** Writing – review & editing, Writing – original draft, Visualization, Validation, Methodology, Investigation, Formal analysis, Data curation, Conceptualization. **Omar Alfandi:** Validation, Supervision, Funding acquisition. **Mohammad Tubishat:** Writing – review & editing, Writing – original draft, Validation.

## Declaration of competing interest

The authors declare that they have no known competing financial interests or personal relationships that could have appeared to influence the work reported in this paper.
